# Estradiol Regulates Expression of Estrogen Receptor ERα46 in Human Macrophages

**DOI:** 10.1371/journal.pone.0005539

**Published:** 2009-05-18

**Authors:** Amy J. Murphy, Paul M. Guyre, Charles R. Wira, Patricia A. Pioli

**Affiliations:** 1 Department of Physiology, Dartmouth Medical School, Lebanon, New Hampshire, United States of America; 2 Department of Microbiology and Immunology, Dartmouth Medical School, Lebanon, New Hampshire, United States of America; University of California Merced, United States of America

## Abstract

**Background:**

Monocytes and macrophages are key innate immune effector cells that produce cytokines and chemokines upon activation. We and others have shown that 17β-estradiol (E2) has a direct role in the modulation of monocyte and macrophage immune function. However, relatively little is known about the ability of E2 to regulate isoform expression of estrogen receptors (ERs) in these cells.

**Methodology/Principal Findings:**

In this study, we quantify expression of ERα and ERβ in human monocytes and macrophages. We also show for the first time that the N-terminal truncated ERα variant, ERα46, is expressed in both cell types. Promoter utilization studies reveal that transcription of ERα in both cell types occurs from upstream promoters E and F. Treatment with E2 induces ERα expression in macrophages but has no effect on ERβ levels in either cell type. During monocyte-to-macrophage differentiation, ERα is upregulated in a time-dependent manner. Previous studies by our group demonstrated that E2 treatment attenuates production of the chemokine CXCL8 in an ER-dependent manner. We now show that ERα expression levels parallel the ability of E2 to suppress CXCL8 production.

**Conclusions/Significance:**

This work demonstrates for the first time that human macrophages predominantly express the truncated ER variant ERαp46, which is estradiol-inducible. This is mediated through usage of the ERα F promoter. Alternative promoter usage may account for tissue and cell type-specific differences in estradiol-induced effects on gene expression. These studies signify the importance of ERα expression and regulation in the ability of E2 to modulate innate immune responses.

## Introduction

Monocytes are released from the bone marrow into circulation and extravasate into peripheral tissues, where they differentiate into macrophages [1]. As key phagocytes, monocytes and macrophages provide both early recognition of pathogens and a crucial bridge between innate and adaptive immunity. The innate immune system deploys rapid antimicrobial responses to pathogenic challenge and simultaneously instructs the adaptive immune system regarding the nature and context of the infectious threat. Activation of innate immunity is mediated through recognition of distinct molecules that are present on a broad diversity of microorganisms. These pathogen-associated molecule patterns (PAMPs) are recognized by Toll-like receptors (TLRs) that are expressed by monocytes and macrophages. Lipopolysaccharide (LPS), a component of Gram-negative bacterial cell walls, binds to its receptor TLR-4 and activates signaling cascades that result in the elaboration of cytokines and chemokines [2]. Production of these factors is important for the recruitment of other inflammatory effector cells and the activation of adaptive immunity.

Macrophages activate adaptive immune responses by phagocytosing foreign molecules and displaying antigens on their surface for recognition by T lymphocytes. The phenotype and function of tissue macrophages are affected by and uniquely dependent on the cellular milieux to which they are exposed, which include cytokines, chemokines and other biological effector molecules such as steroid hormones. In this regard, recent studies have shown that 17β-estradiol (E2) mediates profound effects on monocyte and macrophage immune function [3].

E2 is the major circulating estrogen in pre-menopausal females and has a direct role in the modulation of innate immune function. Previous studies have shown that E2 attenuates production of pro-inflammatory cytokines including IL-6, TNFα and macrophage inhibitory factor [4], [5], [6]. Moreover, we have shown that expression of the proinflammatory chemokine CXC-motif ligand8 (CXCL8) is also decreased by E2 in monocytes that have been challenged with LPS [7]. CXCL8 is central to the recruitment of neutrophils to sites of inflammation, where they aid in mediating pathogen clearance [8]. As a consequence of their role in microbial destruction, neutrophils also damage tissue at infected sites, leading to the induction of inflammation and the mobilization of immune defense [9].

E2 signals are transduced through estrogen receptors, ERα and ERβ. ERs are ligand-inducible transcription factors and are members of the nuclear hormone receptor family. The ability of ERs to modulate transcription requires the recruitment of co-regulatory proteins to the promoters of estrogen-regulated genes through both direct and indirect interactions. In the classical model of E2 action, binding of E2 to intracellular ERs induces retention of the activated steroid-receptor complex in the nucleus. This complex then augments or represses estradiol-specific gene expression by binding with high affinity to sequence-specific cis regulatory motifs contained within the promoters of target genes (reviewed in [10]). These sequences contain a 13 bp palindromic motif (GGTCAnnnTGACC) referred to as the estrogen responsive element (ERE) [11]. Notably, the ERα promoter contains a half ERE consensus binding site [12]. ERE half sites have been shown to mediate transcriptional regulation of many estrogen-responsive genes, including breast cancer 1 associated ring domain 1 (BARD1) and bone morphogenetic protein-6 (BMP-6) [13], [14].

Although ERα and ERβ arise from separate genes on different chromosomes, they share a high degree of overall homology, particularly in the DNA binding domain [15]. The general structure of the ER consists of an N-terminal activator function-1 domain (AF-1), which is followed by the DNA binding domain, a dimerization domain and the ligand binding / AF-2 domain. The AF domains function as co-regulator binding sites. AF-1 cofactor recruitment is independent of ligand binding and AF-2 cofactor recruitment is dependent on ligand binding [16]. The ERα gene is transcribed from at least six up-stream promoters, which results in mRNA products that differ in their 5′ untranslated regions (5′UTRs) and produce the full-length 66 kDa ERα (ERα66) [17]. Alternative splicing of upstream exons directly into exon 2 results in the AF-1 domain-truncated 46 kDa variant of ERα (ERα46) [18], [19]. A 36 kDa ERα variant (ERα36) lacking both AF-1 and AF-2 domains has also been recently described [20]. Similarly, ERβ is transcribed from at least two additional upstream promoters and alternative splicing leads to at least five protein isoforms (ERβ1-5) [15].

Recent work has begun to characterize expression of ERα and ERβ isoforms in cells and tissues, and these results suggest that differences in the expression of ER isoforms influence gene regulation. For example, the 46 kDa ERα isoform, which has been identified in human endothelial cells [21], osteoblasts [19] and the breast carcinoma cell line MCF-7 [18], has been shown to repress the AF-1 activity of full-length ERα 66 [18]. Furthermore, studies conducted by Metivier et al suggest that ERα46 selectively represses transcription of the E2-responsive pS2 gene through the recruitment of corepressors [22], [23]. This is in marked contrast to full length ERα66, which mediates transcriptional activation of the pS2 gene [23]. Therefore, altered expression of ERα46 vs. ERα66 may account for differential effects of estradiol on gene expression.

Although estradiol has been shown to directly affect monocyte and macrophage immune function, relatively little is known about ERα isoform expression and the ability of estradiol to modulate expression of ER isoforms in these cells. In this study, we demonstrate for the first time that full length ERα66 and the ERα46 splice variant are expressed in primary human monocytes and macrophages. Since previous studies suggest that E2 modulates ER expression in other cell types [24], [25], [26], [27], we asked whether E2 regulates ER expression in monocytes and macrophages. We now show that E2 induces ERα46 in macrophages, but has no effect on ERα expression in monocytes. In contrast, E2 does not regulate ERβ expression in either cell type. Finally, we provide evidence of a direct correlation between ERα expression levels and suppression of LPS-induced CXCL8 secretion. This may be significant for the reduction of tissue damage induced by excessive recruitment of leukocytes, particularly neutrophils, during an immune response. Collectively, these findings indicate the importance of alternative ERα isoform expression and implicate a role for estradiol in the regulation of monocyte and macrophage activation.

## Results

### Expression of ERα and ERβ quantitatively differs in primary human monocytes and monocyte-derived macrophages

**Figure 1:**
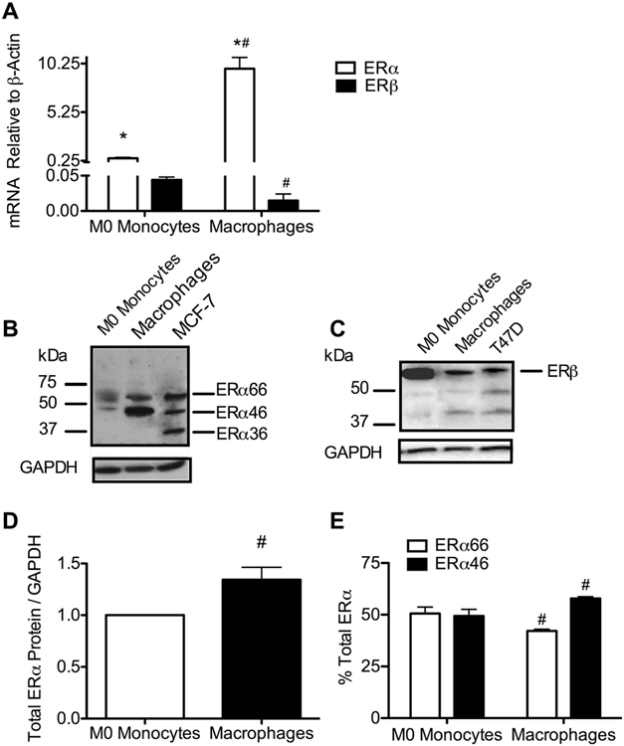
Estrogen receptors are differentially expressed in monocytes and macrophages. *A*, total ERα and ERβ mRNA from M0 monocytes and macrophages from 3–4 individual donors were measured using TaqMan real-time PCR and values were normalized to β-actin. *B* and *C*, expression of ERα and ERβ protein was assessed by western blot analysis. Blots for GAPDH serve as a loading control. *D*, densitometry of total ERα protein expression normalized to GAPDH for three individual donors. *E*, the relative expression of ERα66 and ERα46 expressed as percent total ERα. *, p<0.05 vs ERβ. *#*, p<0.05 vs monocytes.

We and others have reported expression of ERs in human monocytes [7], [28], [29] and macrophages [30], [31], [32]. However, expression of ERα and ERβ had not been quantified in these cell types. Using quantitative Taqman PCR, we demonstrated that ERα mRNA was expressed at higher levels than ERβ in both freshly isolated monocytes (M0) and macrophages that had been differentiated with GM-CSF for 7 days. Although macrophages expressed higher levels of ERα message relative to monocytes, ERβ message levels were lower in macrophages when compared with monocytes (Figure 1A). Consistent with these findings, total ERα protein levels were significantly higher in macrophages, while ERβ protein expression was greater in monocytes (Figure 1B–D).

Three ERα isoforms have been reported: full length ERα (ERα66) and the splice variants ERα46 and ERα36. Consistent with previous reports [20], [33], we were able to detect all three isoforms of ERα in MCF-7 breast cancer cells (Figure 1B). In addition to expression of ERα66, we also detected expression of ERα46 in both human primary monocytes and monocyte-derived macrophages (Figure 1B). Intriguingly, monocytes expressed equivalent levels of both isoforms, whereas macrophages expressed more ERα46 relative to ERα66 (Figure 1E). These data are particularly significant because ERα46 has been shown to function as a repressor of ERα66 transcription [18], [19], [34].

### Estradiol increases ERα expression in macrophages, but not monocytes

**Figure 2:**
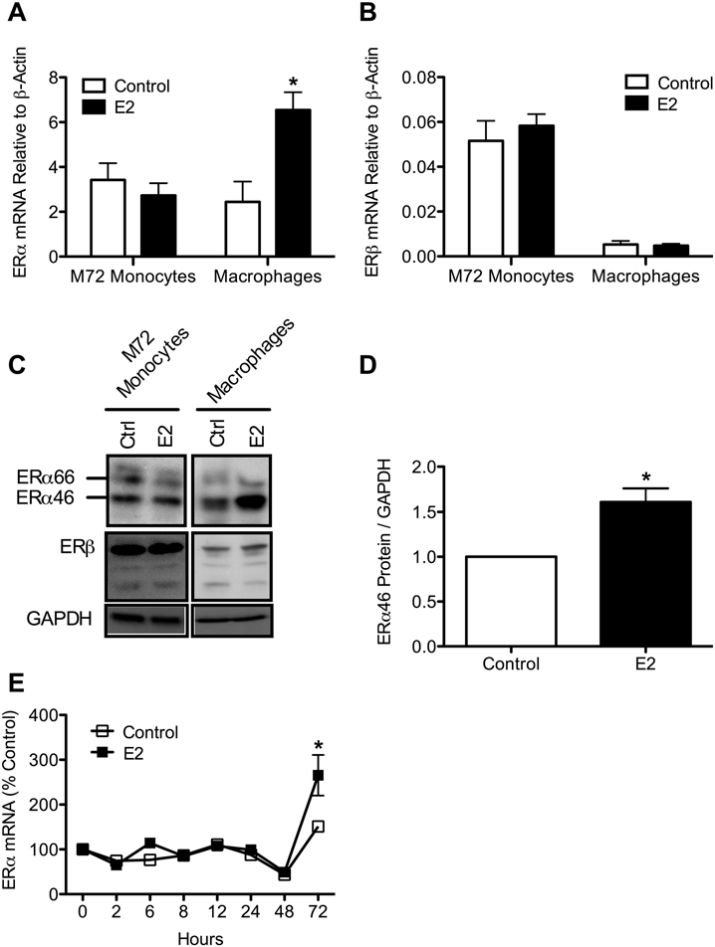
Estradiol induces ERα expression in macrophages and not monocytes. Monocytes (M72) and macrophages were treated with 10^−7^ M E2 or ethanol control for 72 hrs and *A*, ERα and *B*, ERβ mRNA levels were measured. *C*, ERα and ERβ protein were also analyzed. *D*, quantification of macrophage ERα46 expression with and without E2 treatment from three different donors normalized to GAPDH expression. *E*, time course of ERα mRNA expression in macrophages treated with or without E2 normalized to β-actin and expressed as percent control. *, p<0.05 vs. control.

Previous work has demonstrated that E2 modulates ER expression in endometrial carcinoma cells [24]. To determine whether E2 regulates ER expression in monocytes and macrophages, cells were treated with 10^−7^ M E2 for 72 hrs. In this study, freshly isolated monocytes were cultured with estradiol for 72 hours. To distinguish these cells from the freshly isolated monocytes in Figure 1, we refer to these cells as M72. M72 monocytes have been cultured for a full 72 hours longer than the M0 monocytes in Figure 1. Fully differentiated GM-CSF-matured macrophages were also treated with estradiol for 72 hours. Following treatment, M72 monocytes and macrophages were lysed and ER mRNA and protein expression levels were assayed. As demonstrated in Figure 2 (A and C), E2 treatment induced ERα mRNA and protein expression in macrophages, but had no effect on M72 monocyte ERα levels. In contrast, E2 had no effect on ERβ expression in either cell type (Figure 2B and C). The E2-induced increase in total macrophage ERα protein levels can be attributed predominantly to up-regulation of ERα46 (Figure 2C and D). To determine whether E2 influenced ERα mRNA expression at earlier time points, GM-CSF-matured macrophages were treated with E2 or vehicle control, and ERα message was measured over the course of 72 hours (Figure 2E). We observed a small and transient increase at 6 hrs that returned to base line by 8 hrs, followed by a large increase in ERα message at 72 hrs. E2 treatment did not affect cell viability in either M72 monocytes or macrophages as measured by CellTiter-Blue viability assay at any of the time points analyzed (Figure S1).

### ERα increases during monocyte-to-macrophage differentiation

**Figure 3:**
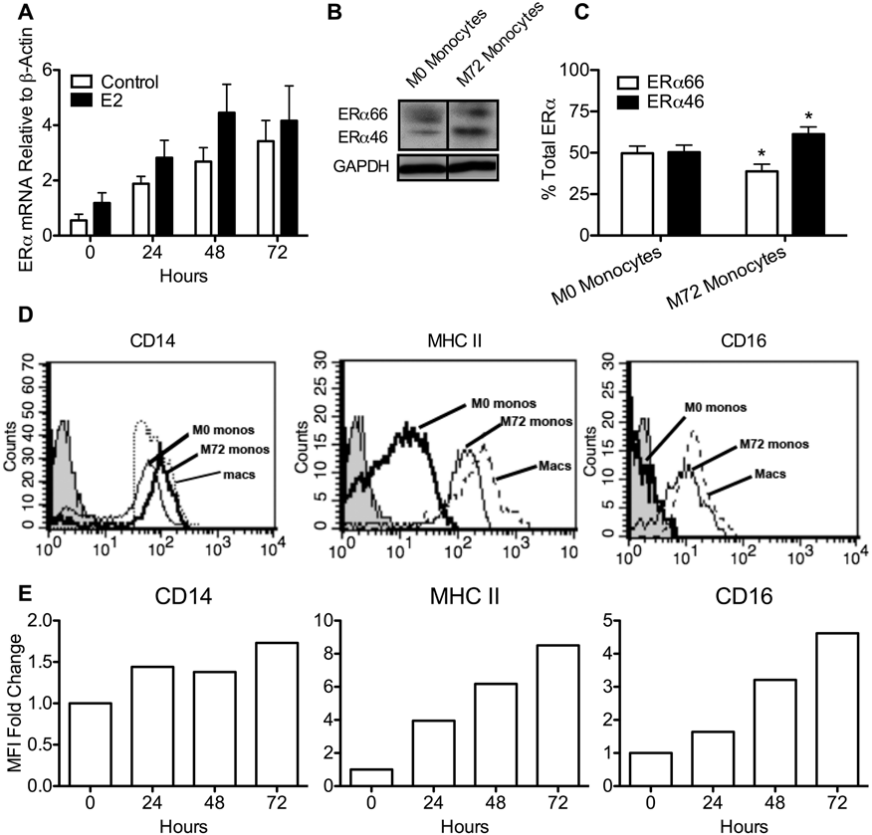
ERα expression increases during monocyte-to-macrophage differentiation. *A*, monocytes were cultured for 0, 24, 48 or 72 hrs with 10^−7^ M E2 or ethanol control and ERα mRNA expression was measured and normalized to β-actin expression. *B*, ERα protein expression of monocytes (M72) cultured for 0 hrs and 72 hrs. *C*, comparison of ERα66 and ERα46 expression at 0 and 72 hrs expressed as percent total ERα. *, p<0.05 compared to monocytes. *D*, flow cytometric analysis of freshly isolated monocytes (M0), monocytes cultured for 72 hours (M72), and monocytes differentiated with GM-CSF for 7 days (macs). Cells were stained for surface expression of CD14, MHC II and CD16. *E*, change in mean fluorescence intensity in monocyte expression of CD14, MHC II and CD16 following 0, 24 hrs, 48 hrs and 72 hrs of culture.

The observation that macrophages express higher levels of ERα mRNA and protein than monocytes (Figure 1) led us examine whether ERα is up-regulated during the differentiation of monocytes to macrophages. To address this question, monocyte ERα mRNA levels were measured during the first three days of cell culture in the absence of added cytokine. We also examined whether E2 treatment during differentiation influenced ERα expression. Monocytes were cultured for 0, 24, 48, or 72 hrs in the presence and absence of E2. As shown in Figure 3A, ERα mRNA expression increased in a time-dependent manner, and this occurred irrespective of E2 treatment. Consistent with this finding, ERα46 protein expression was significantly higher after 72 hrs compared to time 0 (Figure 3B). Moreover, by day 3 of culture, the expression profiles of ERα66 and ERα46 in M72 monocytes reflected those of fully differentiated macrophages (Figure 3C). These data are consistent with our finding that monocytes begin to assume macrophage-like characteristics with increased duration of cell culture (Figures 3D and 3E). To determine the differentiation status of these cells, flow cytometric analysis of key cell-surface markers was performed. Consistent with the findings of Martinez et al. and Lehtonen et al. [35], [36], we observed that expression of CD14, MHC II and CD16 increases as monocytes begin to differentiate into macrophages (compare M0 with M72 monocytes) and that fully differentiated macrophages exhibit higher levels of cell surface CD14, MHC II and CD16 (Figure 3E). These observations illustrate that during monocyte-to-macrophage differentiation, ERα is up-regulated in a time-dependent manner. Furthermore, these studies show that estradiol induces ERα expression only in completely differentiated macrophages, since estradiol treatment failed in increase ERα levels in M72 monocytes (Figure 3A) but did enhance ERα expression in GM-CSF-matured macrophages (Figure 2C).

### ERα is transcribed from upstream promoters E and F in monocytes and macrophages

**Figure 4:**
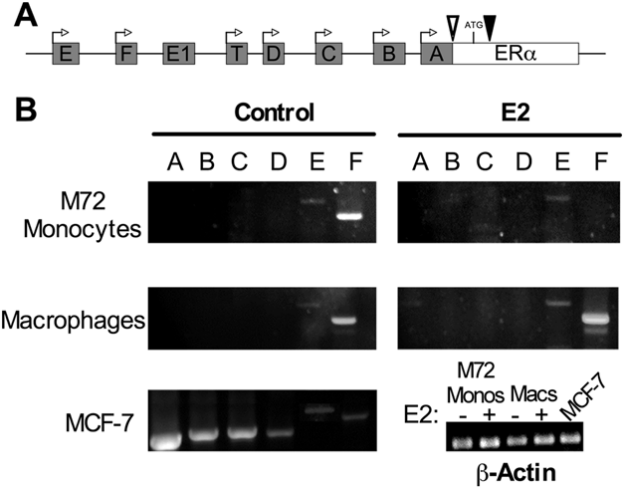
ERα promoter utilization in monocytes and macrophages. *A*, schematic representation of the ERα promoter. Boxes A–F represent up-stream exons and forward arrows denote transcription start sites. The inverted open triangle indicates the primary splice acceptor site and the inverted closed triangle represents the alternative splice acceptor site responsible for ERα46 message. Exons E and F splice into exon E1 before splicing to the ERα coding sequence. *B*, cells were treated as in Figure 2 and RT-PCR analysis was performed using forward primers specific for each upstream exon and a reverse primer specific for exon 2 (see Table 1) to determine ERα promoter usage. Reactions for β-actin were used as a control for cDNA integrity.

Transcription of ERα occurs from at least seven different promoters, resulting in mRNAs that differ in their 5′ UTRs [17] (Figure 4A). Translation of messages produced from all promoters generates full-length ERα66 protein. Alternative splicing of messages transcribed from the distal promoters E and F can also result in production of the ERα46 isoform [12]. Since promoter usage varies among tissues and cell types [16], we investigated which promoters were utilized by monocytes and macrophages and whether they were regulated by E2. To address these questions, primers were designed such that the forward primers were specific for the 5′ UTR of the ERα mRNA produced by transcription from each promoter and the reverse primer was directed towards exon 2 of the coding sequence (Table 1). Each reaction amplified only messages produced from the given promoter analyzed. Using MCF-7 cells as a positive control, RT-PCR analysis confirmed utilization of promoters A–F [16]. Interestingly, both monocytes and macrophages utilize only promoters E and F for the transcription of ERα (Figure 4B). To determine whether E2 influences promoter usage, monocytes and macrophages were treated with E2 for 72 hrs and promoter usage was analyzed. As demonstrated in Figure 4B, treatment of monocytes with E2 resulted in preferential utilization of promoter E vs. promoter F. However, macrophages continued usage of both promoters and appeared to increase transcription from promoter F (Figure 4B). Therefore, the disparity in E2 regulation of ERα expression in monocytes and macrophages may be attributable to differential promoter utilization.

**Table 1:** Primer sequences for promoter utilization studies.

Primer Name	Sequence
Promoter A forward	5′-CATGCGCTGCGTCGCCTCTAA-3′
Promoter B forward	5′-GCCCAGTCTTCCCTGGGC-3′
Promoter C forward	5′-CCACTCGCACATGCGAGCAC-3′
Promoter D forward	5′-GTGGGGCTGGAGACACATTCAACG-3′
Promoter E forward	5′-GCAGTCAGAGAAATAATCGCAGAGCCTCAAATA-3′
Promoter F forward	5′-CATGGTCATAACAGCCTCCTGTCTACCG-3′
ERα exon 2 reverse	5′-GCTCAGCATCCAACAAGGCACTGA-3′
β-actin forward	5′-TCTCTTGCTCTGGGCCTCGTC-3′
β-actin reverse	5′-TCGCCGCGGTCGTCGTC-3′

### Estradiol induction of ERα promoter activity is mediated by a half ERE site

**Figure 5:**
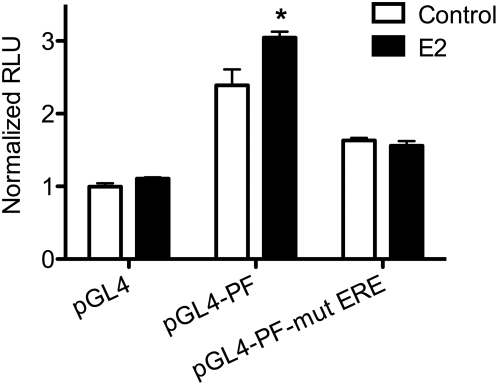
Estradiol induction of ERα promoter activity is mediated by a half ERE site. RAW 264.7 cells were transiently transfected with the pGL4 luciferase expression vector containing either the wild-type ERα promoter F (pGL4-PF) or the half ERE mutated promoter F (pGL4-PF-mut ERE). Cells were treated with 10^−7^ M E2 for 48 hrs and then analyzed for luciferase activity. Results are expressed in relative light units normalized to vehicle-treated empty vector and are representative of at least three independent transfections. *p<0.05 vs. vehicle control.

E2 modulates transcriptional regulation of gene expression through selective binding to EREs, and the ERα F promoter contains a half ERE consensus binding site [11], [12]. To elucidate the role of this half ERE in the E2-mediated induction of ERα46 expression in macrophages, an ERα F promoter luciferase expression vector was constructed that contains a mutation in the half ERE site (−117181 to −117177). RAW264.7 macrophages that endogenously express ERα were transfected with this construct, treated with estradiol and analyzed for ERα promoter activation. As demonstrated in Figure 5, mutating the ERE completely abrogated the ability of E2 to induce ERα F promoter-driven luciferase expression. Intriguingly, mutations in the ERE also inhibited basal ERα transcription (Figure 5). These studies implicate a role for the ERE in mediating estradiol induction of ERα46 expression in macrophages.

### ERα levels mirror attenuation of LPS-induced CXCL8 production

Recognition of PAMPs by TLRs activates monocytes and macrophages to produce cytokines and chemokines. LPS binding to TLR4 induces production of CXCL8, which attracts neutrophils to sites of inflammation. Neutrophils aid in pathogen clearance via phagocytosis and the release of proteolytic enzymes. In this regard, neutrophils are beneficial in resolving the infection. However, excessive neutrophil infiltration can cause significant tissue damage [8].

**Figure 6:**
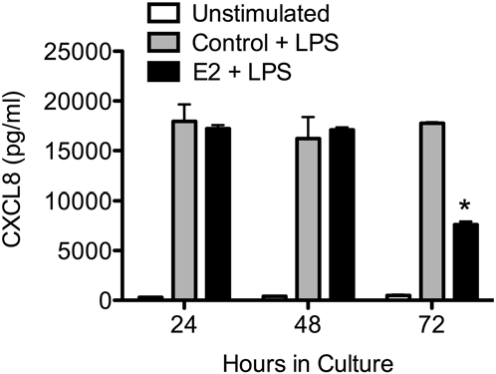
ERα expression mirrors E2 modulation of LPS-induced CXCL8 production. Monocytes were cultured for 24, 48 or 72 hrs. 10^−7^ M E2 or ethanol control was added during the final 24 hrs of culture. Subsequently, cells were stimulated in the presence or absence of LPS (10 ng/ml), for an additional 12 hrs. Culture supernatants were analyzed for CXCL8 by ELISA and reported as pg/ml. *, p<0.05 vs. control + LPS.

Previously, we have demonstrated that E2 attenuates LPS-induced CXCL8 production and subsequently reduces neutrophil chemotaxis [7]. To test the hypothesis that ERα expression levels directly contribute to the ability of E2 to suppress CXCL8, monocytes were cultured for 24, 48 or 72 hrs to allow for time-dependent increases in ERα expression. During the final 24 hrs of culture, E2 or vehicle control was added to the culture medium. The cells were then stimulated or not with 10 ng/ml LPS for 12 hrs. Culture supernatants were analyzed for CXCL8 production by ELISA and as expected, at all three time points LPS induced robust production of CXCL8 compared with unstimulated cells (Figure 6). There were no significant differences in CXCL8 production in 24 or 48 hr cultures whether they were treated with E2 or not. Importantly, monocytes cultured for 72 hrs and treated with E2 produced significantly lower levels of CXCL8 compared with vehicle control (Figure 6). As we have demonstrated previously, attenuated CXCL8 production is not attributable to impaired cell viability, as estradiol treatment does not induce apoptosis in M72 monocytes [7]. These findings suggest that expression of the ERα46 isoform is coincident with attenuation of LPS-induced CXCL8 production.

## Discussion

Many studies have reported direct modulation of innate immune function by E2; however, the isoform expression profile and regulation of ERs in monocytes and macrophages were unknown. Here we report differential ERα isoform expression in primary human monocytes and monocyte-derived macrophages. In addition to the full-length ERα66, we demonstrate for the first time that the ERα46 variant is expressed in both cell types. Promoter usage studies revealed that transcription of ERα occurs from the upstream promoters E and F in monocytes and macrophages. In addition, E2 treatment induced ERα expression in macrophages, but had no effect on monocyte ERα levels. E2 had no effect on ERβ expression in either cell type. During monocyte-to-macrophage differentiation, ERα levels increased in a time-dependent manner. Functional studies provide a direct relationship between ERα expression level and E2 suppression of LPS-induced CXCL8 production. Taken together, these data highlight the role of ERα expression and regulation in E2 modulation of the innate immune response to endotoxin challenge.

Our current findings show that expression of ERα is greater than ERβ in both monocytes and macrophages. Macrophages express higher levels of ERα and lower levels of ERβ than monocytes. Although ERα and ERβ share a high degree of homology and ligand specificity, their gene targets differ substantially [35]. In fact, many immune effects attributed to E2 in monocytes and macrophages are thought to be mediated through ERα and not ERβ [36], [37], [38], [39].

The ERα variant, ERα46, was identified in both cell types along with the full-length ERα66. Intriguingly, monocytes and macrophages also differ in the pattern of ERα66 and ERα46 expression. Monocytes express equivalent levels of the two proteins, while in macrophages ERα46 is more highly expressed than ERα66. Osteoblasts are another cell type that, like the monocyte lineage, also arise from CD34^+^ hematopoietic progenitor cells [40]. Interestingly, osteoblasts also express both ERα66 and ERα46 [19]. Another innate immune cell type, the dendritic cell, differentiates from monocytes and shares some overlapping immunological function with macrophages. In a recent publication, Nalbandian et al. demonstrate that blocking ERs impairs proper dendritic cell differentiation [41]. These findings, in conjunction with our observation that ERα is up-regulated during macrophage differentiation, suggest an important role for ER signaling during the differentiation of innate immune cells.

The organization of the ERα gene is complex, with at least six distal promoters described to date [17]. Splicing of upstream exons prior to the first coding exon results in messages that differ in their 5′ UTRs, and translation of these messages produces full length ERα66. Alternative splicing of upstream exons E and F directly into exon 2 of the ERα gene is responsible for production of the ERα46 isoform. Using RT-PCR, we demonstrated usage of both promoters E and F in monocytes and macrophages, consistent with expression of ERα66 and ERα46. Although little is known about auto-regulation of ERα, two promoters have been previously identified as E2-inducible: promoters C [27] and F [42]. We demonstrated that macrophages preferentially use promoter F and that E2 treatment augments total ERα mRNA and ERα46 protein expression. However, in monocytes E2 had no effect on promoter E usage, but abrogated the usage of promoter F. These findings allow us to conclude that through distinctive changes in promoter usage with E2 treatment, macrophages are able to up-regulate ERα expression with E2 treatment, whereas monocytes do not.

One possible explanation for this observation could be that since macrophages express higher levels of ERα, E2 signaling may be enhanced in these cells, thereby allowing for ERα auto-regulation. In this study, we have shown that estradiol enhances ERα F promoter activity and have identified a half ERE site as the key mediator of this induction. This is in accordance with the findings of Lambertini et al, who demonstrated a key role for the F promoter half ERE site in estradiol-mediated induction of ERα in human osteoblasts [43]. Intriguingly, mutation of the F promoter ERE also inhibited basal transcription in the absence of hormone treatment (Figure 4). In this regard, recent work has shown that unliganded ER acts as a transcriptional co-activator through association with the transcription factors *c-jun* and NFκB [44]. Therefore, it is possible that ERα activates transcription through association with co-activators while it is bound to the ERE and can no longer associate with these proteins when the ERE is mutated, resulting in transcriptional repression. Alternatively, unliganded ERα may complex with other proteins, bind different transcriptional regulatory sites and inhibit basal transcription. Notably, ERs have been shown to modulate gene expression in the absence of ERE binding through association with transcription factor binding elements such as CRE-D1 [45].

Time course studies in macrophages over a period of 72 hrs showed a small and transient increase in ERα at 6 hrs E2 treatment; however, the major induction of ERα mRNA occurred at 72 hrs. The time frame in which we observed induction of ERα suggests an intermediate factor regulated by E2 activates ERα expression. One potential intermediate is the homeodomain transcription factor BARX2. Over-expression of BARX2 resulted in a 2-fold increase in ERα66 and a 5-fold increase in ERα46 in MCF-7 cells [46]. Moreover, there is a homeobox binding site directly upstream of the half ERE in the ERα F promoter (our unpublished observation). Although it is unknown whether E2 modulates BARX2 expression, there are multiple elements within the BARX2 promoter that suggest potential regulation by E2. Notably, there are several EREs within 1 kb upstream of the BARX2 gene, in addition to binding sites for AP-1 and SP-1 transcription factors (our unpublished observations). It is possible that E2 up-regulates BARX2, which in turn induces ERα transcription. This could occur with BARX2 acting alone or in concert with ERα binding to the half ERE site in the F promoter [46].

In this regard, our findings demonstrating the importance of timing of E2 treatment are consistent with recent studies conducted by Calippe et al., who have shown in murine macrophages that short vs. long-term exposure to estradiol mediates differential effects on cytokine production [47]. Our current work demonstrates that treatment with E2 for 6 hours induced a transient elevation in ER expression in human macrophages, but it was only after a 72 hour incubation with E2 that ER expression levels were markedly elevated. These results suggest that induction of ER expression was not observed in monocytes but was seen in macrophages because of the timing required for this E2-mediated event.

Exposure to E2 can alter the innate immune response to infection. Recent work has shown that E2 decreases production of multiple cytokines and chemokines in response to inflammatory signals [4], [5], [6], [7] and gender-based studies in humans have established sexual dimorphisms in immune responses. For example, pre-menopausal females have a lower incidence of trauma related sepsis in comparison to males [39]. Moreover, post-menopausal women have an increased sepsis mortality rate compared to pre-menopausal women [3], [39]. These findings imply that hormonal modulation of innate immune function may play a role in mediating these effects. We now show that after 72 hrs of culture, the ERα expression profile of monocytes changes to that of fully differentiated macrophages, in which ERα46 is the predominant receptor isoform. Significantly, E2 pretreatment of these cells attenuated LPS-induced CXCL8 production. Together, these observations designate a role for sex hormones in modulating immune responses.

In initial studies, we demonstrated that estradiol modulates ERα expression in a dose-dependent manner. We chose to perform the remainder of our work with 10^−7^M estradiol because it demonstrated the maximal effect on ERα46 induction in macrophages. This concentration is physiologically relevant because it is consistent with levels present in the human female reproductive tract during ovulation. Estradiol levels in the ovarian vein, which drains directly into the human female reproductive tract, are 14–80 fold higher during ovulation than levels measured in peripheral blood, and more than 100-fold higher than during the early proliferative phase of the menstrual cycle [48]. Estrogen has been shown to accumulate in the cycling human uterine endometrium and vagina to at least 37 and 11 times that seen in plasma, respectively [49]. Therefore, macrophages at these sites are routinely exposed to this concentration of estradiol. Intriguingly, 10^−7^M estradiol also has physiologic relevance during pregnancy [50], [51], and thus our studies may have significant immunologic relevance at this time.

ERα46 is a functional E2 receptor that can form homodimers as well heterodimers with ERα66 and ERβ. Intriguingly, ERα46 homodimers have a higher affinity for the ERE than ERα66 homodimers. Transcriptional activation of genes that are induced by E2 and dependent on AF-1 domain cofactor binding are repressed in systems where ERα46 is over-expressed, indicating that ERα46 can block ERα66-mediated transcription [18], [34]. Previously, we have shown that E2 modulation of CXCL8 production occurs via transcriptional inhibition [7]. Given these data, it is possible that ERα46 mediates suppression of CXCL8 transcription via suppression of ERα66. Although the role of ERα46 in regulating immune function has yet to be defined, these studies suggest the importance of alternative ERα isoforms in regulating monocyte and macrophage responses to endotoxin challenge.

Furthermore, differential expression of ERs in monocytes and macrophages may account for the well-documented variations in the effects of estradiol in these and other cell types. The ability of estrogen to transduce signals may be dependent on the ratio of cellular ER isoform expression. In this model, estradiol selectively enhances or represses gene expression based on the dominant ER isoform expression and profile of cofactors present in the cell. Thus, estradiol will have different effects on cell types in which full-length ERα predominates vs. the ERα46 splice variant. The influence of these isoforms on cytokine gene expression in monocytes and macrophages is under current investigation in our laboratory.

Here we report, for the first time, the expression of the ERα46 splice variant in human monocytes and macrophages. Furthermore, we demonstrate that estradiol regulates ERα expression in macrophages and show that increased expression of ERα46 is concomitant with the ability of estradiol to attenuate CXCL8 expression in response to endotoxin. As CXCL8 is a potent chemo-attractant for neutrophils, suppression of this chemokine by E2 may be important in reducing excess neutrophil infiltration during an immune response and thereby reducing tissue damage. This may be of particular importance in tissues subject to physiologically high levels of E2, such as the female reproductive tract. In fact, CXCL8 expression in these tissues is repressed when E2 levels are elevated [52] and excessively high levels of CXCL8 correlate with infertility [53], [54]. The studies presented here therefore have broad clinical implications and suggest a role for E2 in the modulation of immune responses during an endotoxin challenge.

## Materials and Methods

### Isolation and culture of human peripheral blood monocytes and macrophages

Peripheral blood mononuclear cells were obtained by leukapheresis of normal, healthy pre-menopausal female donors following informed consent. Written consent was obtained from all subjects in accordance with the human experimentation guidelines established by Dartmouth College's Committee for the Protection of Human Subjects (CPHS), protocol #17011. To preclude confounding results associated with exogenous hormone use, individuals using hormonal contraceptives were excluded from these studies. Mononuclear cells were separated on Ficoll-Hypaque and enriched for monocytes using cold aggregation [55]. This methodology is based upon the ability of mononuclear cells to spontaneously form large cellular aggregates at 4°C and has been used extensively to purify human monocytes [7], [56], [57]. Monocyte purity was >98% as determined by CD14 expression using flow cytometry (data not shown). Freshly isolated monocytes are designated as M0 in the text. In some experiments, monocytes were cultured in the absence of cytokine for 72 hours and treated or not with hormone. These cells are indicated in the text as M72 monocytes. To generate macrophages, monocytes were cultured in the presence of 10 μg/ml GM-CSF (Peprotech) for 7 days. Both human monocytes and macrophages were cultured in complete HEPES-buffered RPMI 1640 (Cellgro) supplemented with 10% FBS (Hyclone) and 50 μg/ml gentamicin sulfate (Sigma-Aldrich).

### Cell lines and cell culture

The MCF-7 and T47D breast cancer cell lines (ATCC) and RAW264.7 cells were cultured in HEPES-buffered RPMI 1640 (Cellgro) supplemented with 10% FBS (Hyclone) and 50 μg/ml gentamicin sulfate (Sigma-Aldrich).

### Estradiol and LPS treatments

Prior to hormone treatment, culture media was switched to HEPES-buffered phenol red-free RPMI 1640 (Cellgro) supplemented with 10% charcoal dextran-stripped FBS (Hyclone) and 50 μg/ml gentamicin sulfate. Phenol red-free media were used to exclude estrogenic effects. 17β-estradiol (Calbiochem) was resuspended in ethanol immediately prior to treatment. Cells were treated with 10^−7^ M E2 or ethanol as a vehicle control for the indicated duration. For LPS stimulation experiments, cells were pre-treated with E2 for 24 hrs followed by administration of 10 ng/ml *E. coli* LPS (Sigma-Aldrich) for 12 hrs. Culture supernatants were analyzed for CXCL8 production using the human CXCL8 Quantikine ELISA kit (R&D Systems) according to the manufacturer's protocol.

### RNA extraction and RT-PCR

Total RNA was extracted from cells using RNeasy Mini kit with on-column DNase I treatment (Qiagen). RNA integrity and concentration were determined using the RNA6000 Nano LabChip kit (Agilent). 500 ng of RNA were reverse transcribed using the SuperScript III First-Strand Synthesis System for RT-PCR (Invitrogen) according to the manufacturer's protocol. For analysis of ERα transcripts, PCR was performed with *Taq* DNA polymerase (Invitrogen) for 30 cycles using the primers listed in Table 1. Cycling conditions were as follows: an initial 2 minute denaturation at 95°C followed by 30 cycles consisting of 30 sec at 95°C, 30 sec at 57°C, and 1 min per kb DNA amplified at 72°C. A final extension for 10 min at 72°C concluded the reactions. Reactions without reverse transcriptase or without template served as controls for genomic DNA contamination. 10 μl of PCR product was analyzed by electropheresis on 1% agarose gels and photographed under UV light.

### TaqMan PCR

Real-time TaqMan PCR was used to quantify mRNA expression of ERα and ERβ. 0.5 μl of cDNA was added to TaqMan Master Mix (Applied Biosystems) in accordance with the manufacturer's instructions. TaqMan validated primer/probe sets for ERα and ERβ (Applied Biosystems) directed toward the exon 3–4 and exon 4–5 boundaries, respectively, were used to amplify target genes. A β-actin primer/probe set (Applied Biosystems) was used as an internal control to normalize experimental gene expression. In previous studies, we have shown that estradiol treatment does not affect β-actin expression in human monocytes and macrophages [7], [58]. Amplification was carried out using the Applied Biosystems 7300 Real-Time PCR system. Threshold cycle number was determined using Opticon software and ER levels were normalized to β-actin levels using the formula 2^− (Et−Rt)^ where Rt is the mean cycle threshold for the control gene and Et is the mean threshold for the experimental gene. Cycling conditions for TaqMan PCR consisted of an initial incubation at 50°C for 2 min and 95°C for 10 min followed by 40 cycles of 95°C for 15 sec and 60°C for 1 min. Product accumulation was measured during the extension phase and all samples were run in triplicate.

### Preparation of lysates and immunoblot analysis

Whole cell lysates were prepared using M-PER mammalian protein extraction reagent (Pierce) according to the manufacturer's instructions. Lysates were analyzed for total protein concentration using a Micro BCA Protein Assay kit (Pierce). 25 μg of each lysate were separated on 10% acrylamide gels and electrotransferred to nitrocellulose membrane in Tris-glycine buffer with 20% methanol. Membranes were blocked in 5% milk in 1X TBS and 0.1% Tween-20 for 1 h at room temperature. Blots were then probed with either mouse mAb TE111.5D11 (Abcam) for ERα or mouse mAb ab16813 (Abcam) for ERβ detection, followed by goat anti-mouse HRP-conjugated secondary antibody (BioRad). To control for protein loading, blots were probed for GAPDH expression using mouse mAb 6C5 (American Research Products). Incubations with primary antibody were overnight at 4°C with rocking and incubations with secondary antibody were performed at room temperature for 45 min following thorough washing. Blots were visualized using Supersignal chemiluminesence substrate (Pierce). Images were scanned and signal density was quantified using the ChemiImager 5500 software (Alpha Innotech).

### Flow cytometric analysis

Surface expression of CD14, MHC II and CD16 were assessed using CellQuest analysis software on a FACScan (BD Biosciences) flow cytometer. Macrophages and monocytes that were cultured as indicated in Figure 3 were incubated with normal human IgG (6 mg/ml) to block FcR-specific binding of mAbs and 40 μg/ml of FITC conjugated antibodies AML-223 (anti-CD14), L243 (anti-MHC II), or 3G8 (anti-CD16). The cells were washed and fixed in 1% MFF. Mean fluorescent intensity (MFI) was determined by the geometric mean of fluorescence of the cells and unstained cells served as a negative control.

### Luciferase Reporter Plasmid Construction

The ERα F promoter was PCR amplified from human genomic DNA using the forward primer 5′CCTCTGTACTGGGTACTGGGAC3′ and reverse primer 5′CTTGAAGAGAAGATTATCACTCAGAGACTGTCT3′ and cloned into the pCR2.1-TOPO vector using the TOPO TA Cloning kit (Invitrogen). The F promoter was then sub-cloned into pGL4 luciferase reporter vector (Promega) by digestion with *Kpn*I and *Xho*I. Mutations were introduced using the QuickChange site-directed mutagenesis kit (Stratagene) as per the manufacturer's recommendations. The 1/2 ERE was mutated from 5′GGTCA3′ to 5′GGGCC3′ using the primer set 5′GCATTTCCTAATTTCATGGGCCTAACAGCCTCCTGTCTACC3′ and its general compliment. All plasmids were sequenced prior to transfection.

### Transient Transfection and Luciferase Assay

RAW264.7 cells were plated 24 hours before transfection at a density of 2×10^5^ cells per well in 24-well tissue-culture dishes. Cells were transfected using Lipofectamine 2000 (Invitrogen). Transfection efficiency was normalized to Renilla activity by cotransfection of 40 ng pRL-TK expression vector (Promega) and transfection efficiencies typically ranged from 85%–90%. Cells were incubated with 80 μg reporter plasmid, treated with 1×10^−7^ estradiol 18 hours post-transfection and harvested with passive lysis buffer (Promega). Luciferase activity was determined using the dual-luciferase reporter assay system (Promega) and the Berthold Centro LB960 luminometer. Data were analyzed in triplicate and are represented as normalized relative light units (RLUs).

### Statistical analysis

Results are presented as mean+/−SE. Statistical analysis was performed using a paired Student's *t-* test and significance was achieved at p<0.05.

## Supporting Information

Figure S1 Monocyte viability is not effected by E2 treatment. Monocytes were cultured for 24, 48 or 72 hrs in the presence or absence of 10-7M E2. Viability was measured using the CellTiter Blue assay and data are represented as percent control.
(0.92 MB TIF)
