# Supplementary figures and images for: Estradiol Regulates Expression of Estrogen Receptor ERα46 in Human Macrophages
Source: PLoS One. 2009 May 18;4(5):e5539. doi: 10.1371/journal.pone.0005539 (PMC2678254; doi:10.1371/journal.pone.0005539)

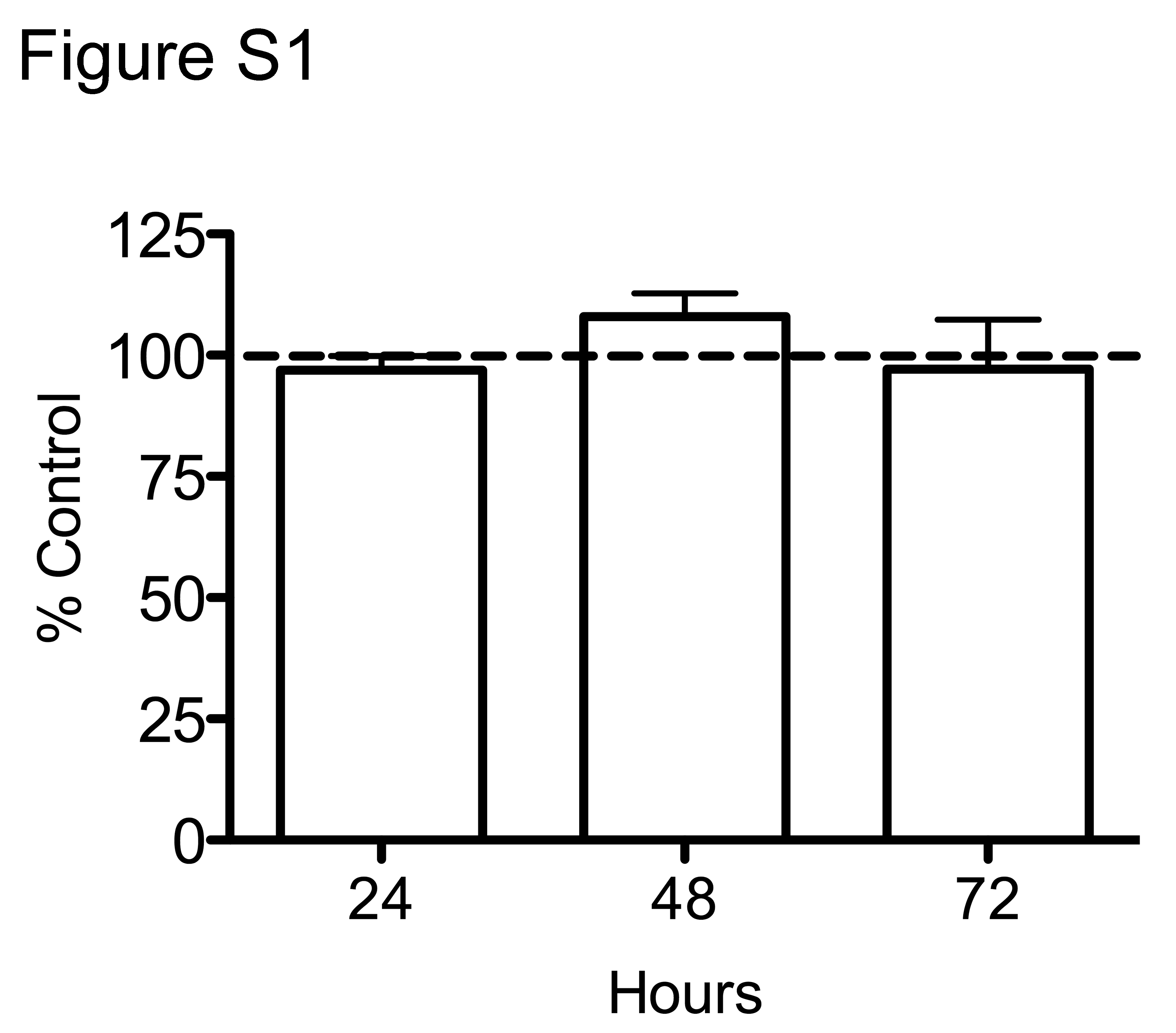

Supplement: Figure S1 — Monocyte viability is not effected by E2 treatment. Monocytes were cultured for 24, 48 or 72 hrs in the presence or absence of 10-7M E2. Viability was measured using the CellTiter Blue assay and data are represented as percent control. (0.92 MB TIF) [file pone.0005539.s001.tif]
